# Supplementary material for: A reference single-cell transcriptomic atlas of human skeletal muscle tissue reveals bifurcated muscle stem cell populations
Source: Skelet Muscle. 2020 Jul 6;10:19. doi: 10.1186/s13395-020-00236-3 (PMC7336639; doi:10.1186/s13395-020-00236-3)
Supplement: Supplementary file 1 — Additional file 1: Figure S1.Comparison of scRNA-seq integration and batch correction methods. We compared four scRNA-seq data integration methods to evaluate which most faithfully conserves donor, anatomical, and biological information while minimizes technical biases. (A) The n = 10 donor datasets were first annotated independently using a nomenclature of 12 common cell type terms following unsupervised SNN clustering. Then we evaluated the integration method by UMAP and by coloring the data either by cell type, donor ID, or 10X library chemistry used. First, we integrated the data by merging the individually normalized gene expression matrices without any further correction. We saw strong technical biases that overwhelmed biological information as the different cell populations segregate by sample/donor and chemistry type. For instance, the two MuSC and progenitor subpopulations are grouped with fibroblasts and endothelial cells. Second, we tested the Seurat SCT integration method [14] . This method first calculates a cross-correlation subspace from genes that are shared between datasets. We noticed that this method better “aligns” donor and chemistry type but at the expense of masking biological variability. For instance, we observed that the two MuSC and four stromal subpopulations (Fibroblast 1,2,3 and Adipocytes) were grouped together, hiding important biological heterogeneity. Although certainly useful to validate reproducibility in scRNA-seq experiments, the Seurat SCT integration approach overcorrected biological heterogeneity for heterogeneous samples. Third, we tested the Scanorama method [13], which relies on a computer vision algorithm that “stitches” datasets together even when the cell type composition between dataset is considerably different. We see that this method groups similar cell populations together while acknowledging donor differences. Yet, surprisingly, this method is also very sensitive at picking up differences in chemistry. To correct [file 13395_2020_236_MOESM1_ESM.docx]

**
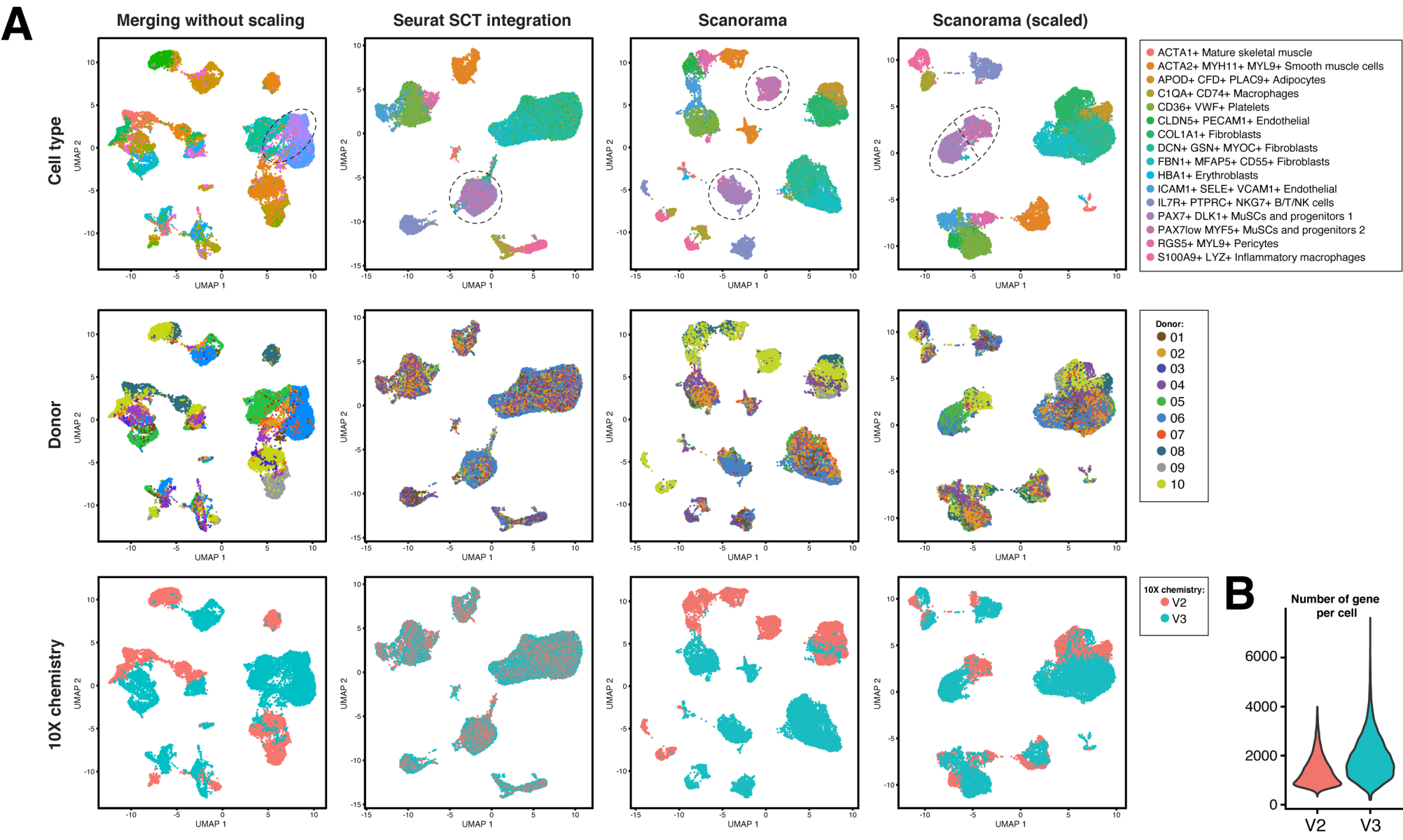
**

**Figure S1.** (previous page) **Comparison of scRNA-seq integration and batch correction methods.** We compared four scRNA-seq data integration methods to evaluate which most faithfully conserves donor, anatomical, and biological information while minimizes technical biases. **(A)** The n = 10 donor datasets were first annotated independently using a nomenclature of 12 common cell type terms following unsupervised SNN clustering. Then we evaluated the integration method by UMAP and by coloring the data either by cell type, donor ID, or 10X library chemistry used. *First*, we integrated the data by merging the individually normalized gene expression matrices without any further correction. We saw strong technical biases that overwhelmed biological information as the different cell populations segregate by sample/donor and chemistry type. For instance, the two MuSC and progenitor subpopulations are grouped with fibroblasts and endothelial cells. *Second*, we tested the Seurat SCT integration method (Stuart et al., 2019b). This method first calculates a cross-correlation subspace from genes that are shared between datasets. We noticed that this method better “aligns” donor and chemistry type but at the expense of masking biological variability. For instance, we observed that the two MuSC and four stromal subpopulations (Fibroblast 1,2,3 and Adipocytes) were grouped together, hiding important biological heterogeneity. Although certainly useful to validate reproducibility in scRNA-seq experiments, the Seurat SCT integration approach overcorrected biological heterogeneity for heterogeneous samples. *Third*, we tested the Scanorama method (Hie et al., 2019), which relies on a computer vision algorithm that “stitches” datasets together even when the cell type composition between dataset is considerably different. We see that this method groups similar cell populations together while acknowledging donor differences. Yet, surprisingly, this method is also very sensitive at picking up differences in chemistry. To correct this chemistry effect, we scaled the Scanorama output by regressing out the chemistry and the number of genes detected per cell (significantly different between chemistry type) **(B)**. Using this integration method, we observed clear separation of the independently annotated cell populations. We present the resulting Scanorama-integrated dataset as a “consensus atlas” (see **Fig. 1B-C**) of human muscle that describes donor-to-donor differences while grouping cells that are similar together and removing technical biases.

**
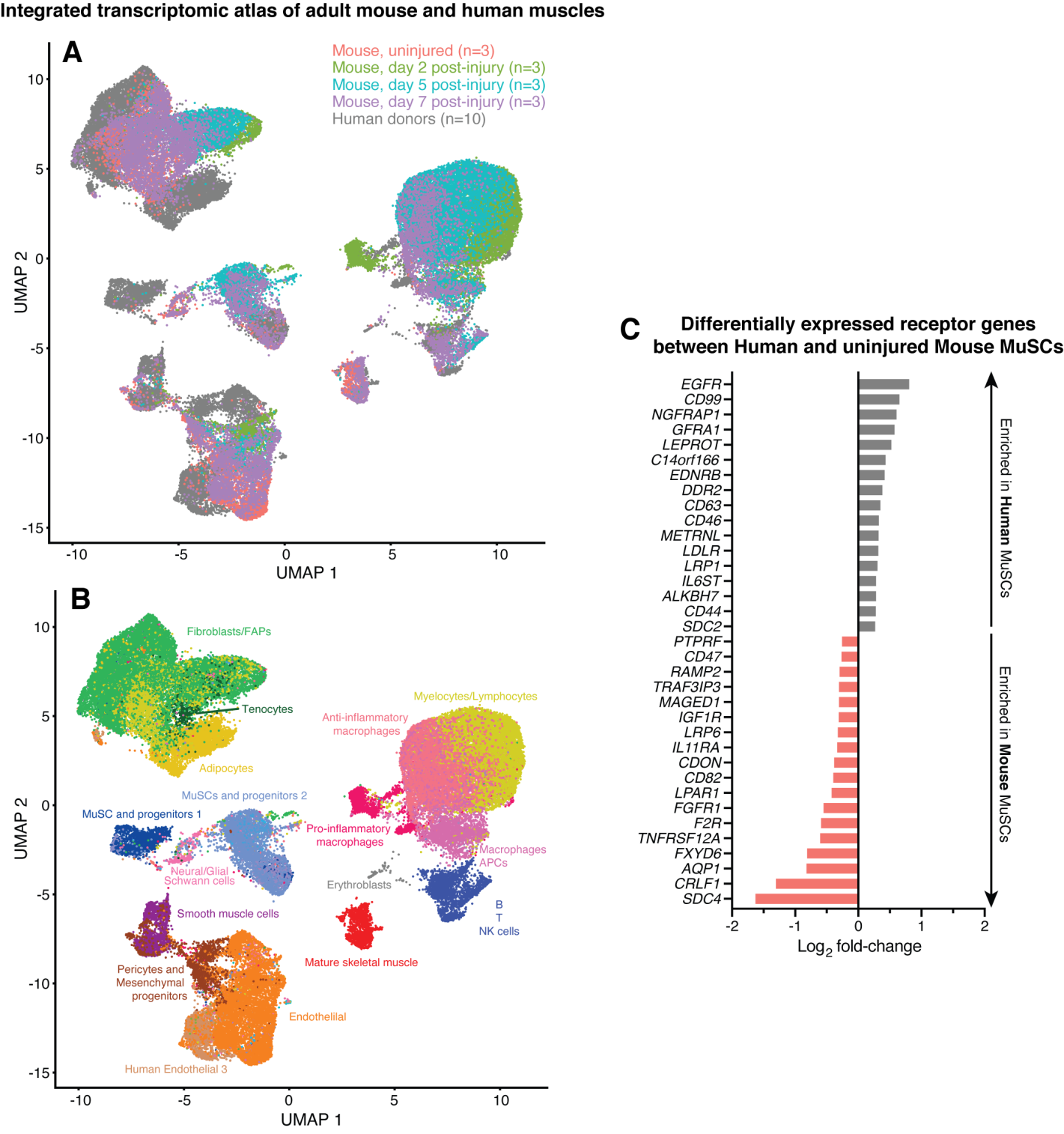
**

**Figure S2.** **Integration of human and mouse scRNA-seq data sets allows comparison of MuSC receptor gene expression across species.** We generated an integrated scRNA-seq atlas including human sample datasets from Fig. 1 and an adult mouse muscle regeneration time-course from De Micheli et al. (2020). These datasets were integrated using first Scanorama and then Harmony for alignment across species. (**A**) Multi-species integrated atlas presented by UMAP plot a colored by sample type. (**B**) Multi-species integrated atlas presented by UMAP plot and annotated by cell-type clusters. (**C**) The human MuSC1 and MuSC2 clusters were grouped into a cumulative human MuSC cell population, which was compared to mouse MuSCs from the uninjured samples only. Receptor genes were analyzed between the mouse and human MuSC cells for differential expression. Differentially expressed genes with an FDR-corrected q-value <0.05 are shown in (**C**).


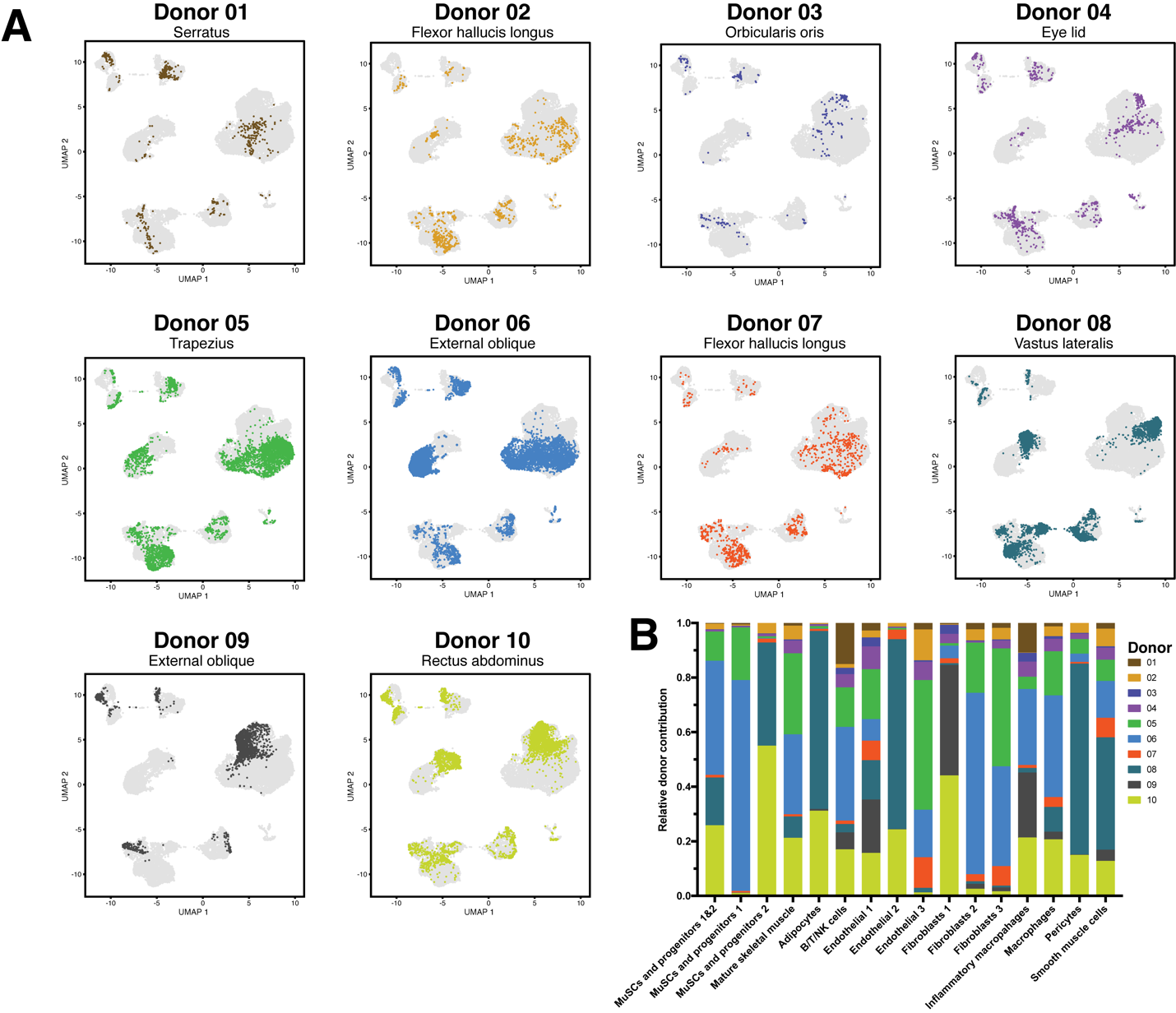


**Figure S3.** **Composition of single-cell reference atlas as a whole and in cell-type clusters by donor. (A)** Visualization of donor (n = 10) contributions to the whole single-cell reference atlas. In each panel, the full atlas is presented as a UMAP plot, with the cells for an individual donor are colored and overlaid on cells from all other donors (in gray). Note the total number of cells assayed differs for each donor (see Fig. 1A). **(B)** Bar plot representing the relative contribution of cells with each cell type cluster from each donor. Note that the MuSC1 and MuSC2 clusters are also plotted as a combined cluster on the left side of the bar plot for reference.


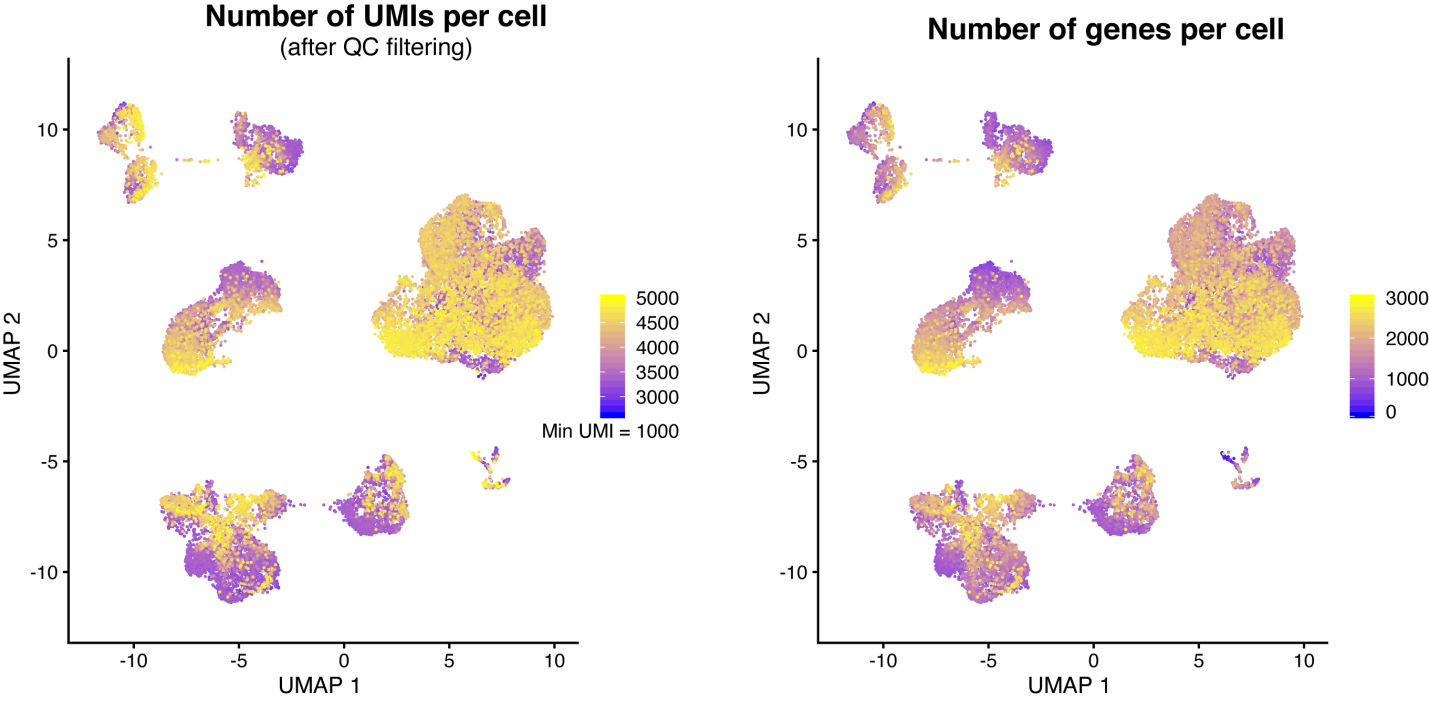


**Figure S4.** **Transcriptomic detection variation within human muscle reference atlas.** UMAP plots featuring (*left*) the number of unique molecular identifiers (UMIs) and (*right*) number of genes detected per single cell. Note that QC filtering removed all cells with less than 1000 UMIs (see Methods).
